# Pharmacovigilance: A Worldwide Master Key for Drug Safety Monitoring: Some Additional Information

**DOI:** 10.4103/0975-1483.80310

**Published:** 2011

**Authors:** C Patel

**Affiliations:** *Department of Pharmacology, Government Medical College, Majuragate, Surat, India*

Sir,

I have read the article titled “Pharmacovigilance: A worldwide master key for drug safety monitoring,” and I found the topic quite informative and it also included recent updates. So first of all, I congratulate the authors for such a nice compilation. It covers almost all areas; however, I would like to add more information about pharmacovigilance in vaccines. The great challenge here is to convey a proper message to the general public as it is like a double-edged sword.[1] Majority of vaccines are administered to vulnerable (children) as well as healthy population that requires strict safety supervision. Therefore, the safety of a vaccination must be more than other pharmacological agents to make it acceptable in general population.[2]

According to WHO, the adverse event following immunization (AEFI) is “a medical incident that takes place after an immunization causes concern, and is believed to be caused by the immunization.” Vaccines are biological agents given prophylactically to protect target population again specific infection by immunological action.[2] Following points favor different pharmacovigilance for vaccines and drugs:

1. Complex vaccine sources

Vaccines are complex biological products, which may include multiple antigens, live organisms, adjuvants, and preservatives. Adverse drug reactions (ADRs) may be due to the administration of live wild viruses, e.g., lymphocyte meningitis after anti-mumps vaccine or may be non-specific, related to a component different from the antigen (aluminum hydroxide involved in the “macrophagic myofasciitis,” allergic reactions to neomycin, latex, egg, or gelatin).[3]

2. Different modes of causality assessment

There are some issues, which make evaluation of vaccines different from other drugs. Local or immediate adverse reactions, which occur due to administrative error and attenuated virus, can be attributed with a degree of confidence but delayed events are difficult to correlate. They have immunological considerations in addition to pharmacological action and take a long time to respond.[3] Criteria commonly used to determine causality such as resolution of the event following treatment discontinuation and the result of rechallenge cannot be used to assess causality of an event occurring after vaccination.[4]

3. Difference between reporters and reporting chain

This suggests big communication gap, which requires immediate modification in the current system.

So, it is high time to change the current strategy for pharmacovigilance of vaccines as considerable research is going on for vaccine development in infectious diseases, e.g., HIV, malaria, H1N1, etc. having high patient load.[2]
